# Extracranial metastases of high-grade glioma: the clinical characteristics and mechanism

**DOI:** 10.1186/s12957-017-1249-6

**Published:** 2017-10-06

**Authors:** Qian Sun, Rui Xu, Hongbo Xu, Gengming Wang, Xueming Shen, Hao Jiang

**Affiliations:** 1 Department of Radiation Oncology, the First Affiliated Hospital of Bengbu Medical College, Bengbu, 233000 China; 2 Department of Surgical Oncology, the First Affiliated Hospital of Bengbu Medical College, Bengbu, 233000 China

**Keywords:** Glioma, Extracranial metastasis, Prognosis

## Abstract

**Background:**

This presentation of two cases and literature review discusses the epidemiology, clinical manifestations, pathogenesis, diagnosis, treatment, and prognosis of high-grade glioma with extracranial metastases.

**Methods:**

A retrospective analysis of the clinical features of two cases of malignant glioma, including metastatic sites, pathological data, and treatment methods, and a literature review was performed.

**Results:**

Two patients developed extracranial metastases within 1 year after surgery for primary glioma. One patient developed cervical lymph node and bone metastases while the other developed bone metastases, and both patients died within 2 months after the diagnosis of the extracranial metastasis.

**Conclusion:**

Extracranial metastases may develop from malignant gliomas. According to the literature, the most common extracranial site is intraspinal (along the neural axis), followed by the vertebrae, lungs, liver, and lymph nodes. The complex metastatic mechanism remains unclear, and the prognosis is very poor, with a survival duration of less than 6 months.

## Background

Malignant glioma is a devastating neurological disease with a uniformly poor prognosis and a clinical course characterized by progressive functional and cognitive impairments [1]. Extracranial metastasis is rare [2], especially in cases without previous surgical disruption of the dura and calvarium, which is thought to seed the extracranial space with tumor cells [3]. Most documented cases of extracranial metastases involve leptomeningeal spread to the spine [4], although metastases to the liver, skin, spleen, lungs, peritoneum, and lymph nodes may also occur [5–8].

High-grade gliomas are particularly insensitive to radiation and genotoxic drugs [9]. Unlike many other malignant tumors of the non-nervous system, the local control of gliomas always fails. Most patients eventually die of local disease progression, recurrence in important areas of the brain, or uncontrollable intracranial hypertension [10].

Surgery is the first-line therapy for glioma. However, gliomas recur in the resection margins of 90% of patients [11]. Many studies have reported that the blood-brain barrier is permissive for monoclonal antibodies (mAbs), both at baseline and in the context of malignancy, thus providing a rationale for treating intracranial malignancies [12] using agents such as bevacizumab [13], anti-CD40 mAbs [14], and anti-CD25 mAbs [15]. Many clinical trials have evaluated mAb-based therapeutic strategies for the treatment of malignant gliomas, and the implementation of such a strategy seems imminent and optimistic [12].

The vast majority of glioma recurrences occur within 2 cm of the primary tumor area [5]; accordingly, the early and correct diagnosis of a tumor recurrence and its differentiation from therapy-related changes is crucial for the prompt and adequate management of glioma patients [16]. Since 2010, our department encountered two cases of extracranial metastasis; here, we discuss these cases along with a review of the literature concerning extracranial metastases of glioma and thus report the epidemiology, clinical manifestations, metastasis distribution, natural history, diagnosis, and treatment of these lesions.

## Methods

### Case 1

**Fig. 1:**
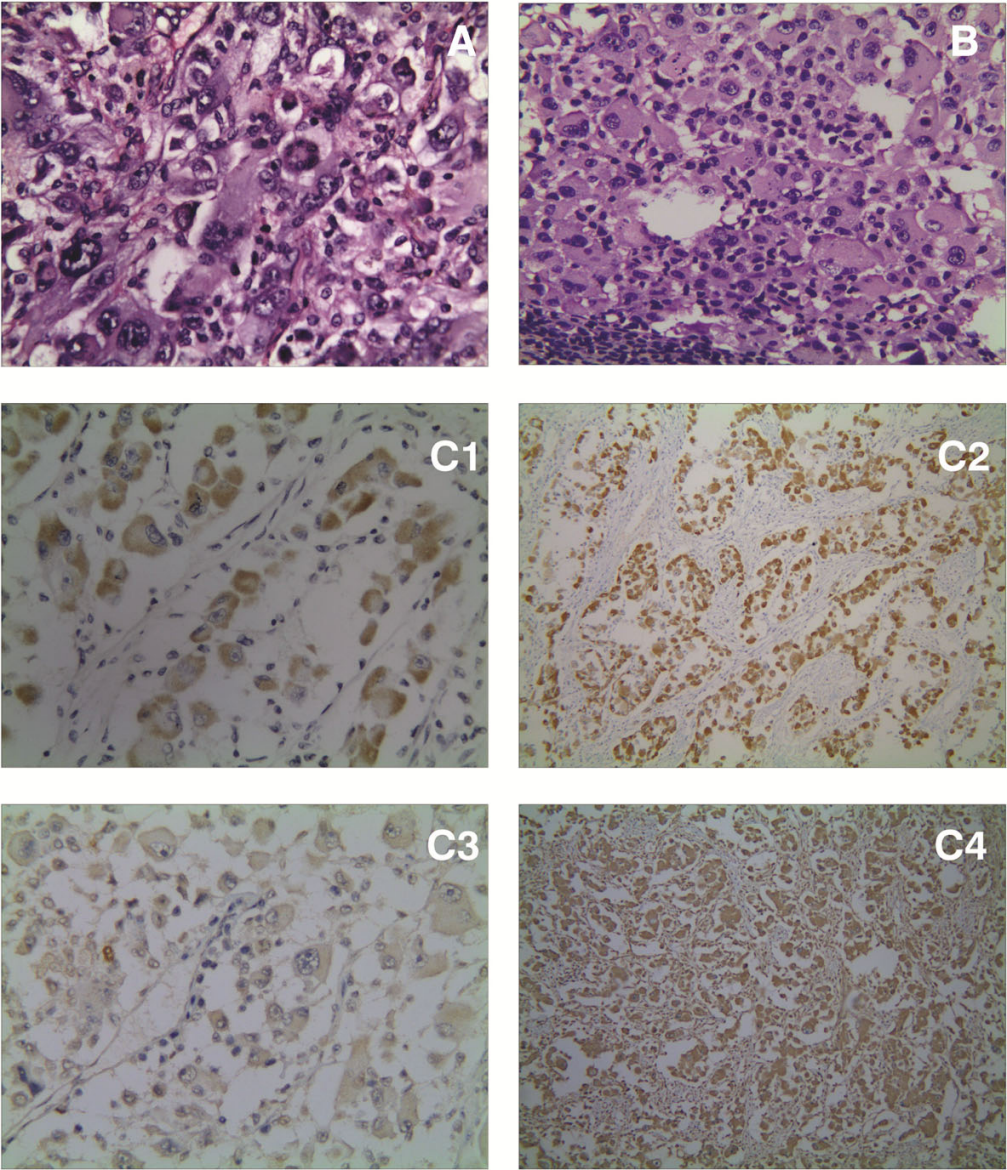
**a** Primary tumor (HE): The pathological section area chosen in A1 picture is untypical. The typical pathological picture with the replaced area has been provided. From the perspective of the new pathological section, it can be observed that primary tumor and metastatic lesion have the consistent histological characters: different cellular size, multinuclear tumor giant cells, obvious nucleolus, red dyeing of cytoplasm, and visible pathological fission. **b** Metastasis of lymphonodus (HE): B1 lymph node metastasis shows lots of lymphocytes. **c** Immunohistochemical results of metastasis (C1: Des; C2: GFAP; C3: S100; C4: Vim)

A 43-year-old man was hospitalized after complaining of intermittent headache and dizziness that progressed in severity for 1 month, as well as an unsteady gait for 2 weeks. Brain magnetic resonance imaging (MRI) revealed high T1 and T2 signals in the left occipital lobe (4 × 3 cm) with peripheral edema and lateral ventricle compression, and a pathological analysis after tumor resection indicated glioblastoma. Although the patient was treated with conformal radiotherapy, metastases of glioblastoma were found in the chest and back, as well as in the left neck (Fig. 1). Figure 1 depicts hematoxylin-eosin (HE)-stained sections of the intracranial primary tumor and metastatic tumor and an immunohistochemically stained section of the metastatic tumor. The HE-stained section of the primary glioblastoma (Fig. 1a) depicts diffusely distributed tumor cells of variable sizes, including giant multinucleated cells, with obvious nucleoli, red-stained cytoplasm, thick nuclear membranes, coarse chromatin, and pathologic mitoses. By contrast, the metastatic sample (Fig. 1b) featured a damaged lymph node structure with only a few remaining lymphocytes. The metastasis comprised mostly tumor tissue, with cells of variable sizes and red-stained cytoplasm. The metastatic tumor cells were eosinophilic, with obvious nucleoli and visible pathologic mitoses. In summary, Fig. 1a, b demonstrates consistent histological characteristics. Figure 1c (C1–C4) depicts an immunohistochemically stained metastatic lymph node specimen. The metastatic tumor was positive for desmin, S-100, glial fibrillary acidic protein (GFAP), and vimentin. The patient exhibited no brain-related neurological symptoms. Emission computed tomography (CT) revealed multiple bone metastases. The patient died 2 months after the detection of the metastatic lesions.

### Case 2

A 41-year-old woman who had undergone tumorectomy 9 months earlier was hospitalized after complaining of pain in the lumbosacral region for 2 weeks. The patient had previously been diagnosed with glioma (World Health Organization (WHO) stage III) in the right temporal lobe and was treated with radiotherapy (total dose, 54 Gy) and intravenous chemotherapy (cisplatin + nimustine, six treatment courses). She had no brain neurological symptoms. Positron emission tomography (PET)-CT revealed a sacral metastasis; however, local radiotherapy (total dose: 10 Gy/5 F) provided no pain relief. The patient refused to continue radiotherapy and died 1 month after the detection of the metastatic lesions. We note that both patients had previously been healthy, with no history of other diseases.

## Results

In this report, we describe two patients who developed extracranial metastases after 1 year: one developed cervical lymph node and bone metastases, and one developed a bone metastasis. Both patients died within 2 months after the extracranial metastases were diagnosed.

## Discussion

Neurosurgeons and oncologists widely believe that malignant gliomas never metastasize outside the central nervous system (CNS). However, this notion has been gradually proven incorrect [17]. Extracranial metastases of malignant gliomas are reported to occur in approximately 0.5% of cases [18]. This low incidence rate may be related to the short lifespans of patients with glioma, or to intrinsic biological obstacles that prevent tumor cells from infiltrating and surviving beyond the neural environment. These obstacles may include (1) the absence of a lymphatic system within the brain and spinal cord that would allow systemic dissemination, (2) the dense dura around intracranial veins that prevents tumor cell penetration, and (3) the lack of nurturing stroma in other organs to facilitate the survival and proliferation of glioblastoma cells [2]. However, increasing numbers of extracranial metastases have been reported in the literature along with advances in therapies for gliomas (e.g., microscopic surgery, radiotherapy, chemotherapy, and other adjuvant therapy) [2, 5].

In the two above-described cases, the surgery for the primary lesion and the diagnosis of the metastases were separated by intervals of 3–9 months, and the lesions were located in the left neck, chest, and sacral bones. According to the literature, the average age of patients at the time of malignant glioma diagnosis was 38–42.2 years [2, 6], the median survival duration is 10.5 months [2], and the interval between the diagnoses of the primary malignant glioma and the extracranial metastasis ranged from 1 to 24 months [2, 6].

Lymph node, vertebral, and sacral metastases were confirmed in both of our patients. The histological types were glioblastoma multiforme (case 1) and anaplastic astrocytoma (case 2). The most common site of extracranial metastasis, according to previous reports, is intraspinal (i.e., along the neural axis) [17], followed by the vertebrae, lungs, liver, and lymph nodes; however, metastases have also been reported in the spleen, scalp, skin, paranasal sinuses, eyes, parotid gland, small intestine, pancreas, kidneys, bone, pleura, and peritoneum [7, 8, 19–22]. Histologically, the primary tumors are usually glioblastoma multiforme, although anaplastic astrocytoma [23], oligodendroglioma [21], gliosarcoma [22], medulloblastoma [24], and pleomorphic xanthoastrocytoma [4] have also been reported. The main pathways of extracranial metastasis include the cerebrospinal fluid shunt, blood, and lymphatic systems [25].

Although the extracranial metastatic mechanism is currently unclear, iatrogenic spread consequent to surgery may be the main cause of extracranial metastases [25, 26]. The two cases in the present study had a history of craniotomy. One patient had been treated with local radiotherapy and systemic chemotherapy after surgery. Metastases of glioma occur along the nerve propagation path [23] or implant in the peritoneum via a ventriculoperitoneal shunt [27, 28]. In addition, radiotherapy can induce a change from glioblastoma to sarcomatoid metaplasia, which can subsequently develop an ability to penetrate vessel walls [29].

Clinical manifestations are based on the sites of extracranial metastases, and symptoms are usually related to the affected organs and tissues. Patients with symptoms and signs in corresponding sites should be subjected to evaluation with an appropriate ancillary modality, such as CT, MRI, single-photon emission CT (SPECT), or PET-CT. MRI is preferred for the evaluation of cases involving symptoms caused by spinal cord or nerve root lesions, MRI and SPECT can be used to evaluate bone pain, and PET-CT may be selected for some cases. If biopsy of the metastases is feasible, tissues from the lesions should be obtained and subjected to pathological examination [30].

A system of diagnostic criteria for extracranial metastases of malignant glioma has been developed. Weiss [31] proposed the following criteria for the diagnosis of extracranial metastases of primary CNS tumors: (1) a clinical history strongly suggesting a primary CNS tumor, (2) pathological findings of metastatic lesions consistent with characteristics of the intracranial primary tumor, although some degree of anaplastic degeneration relative to the primary tumor is acceptable, and (3) performance of a comprehensive autopsy or whole body examination to exclude other primary tumors.

Currently, no standardized protocol exists for the treatment of extracranial metastasis, which is palliative. Most patients receive radiotherapy and/or chemotherapy, and no significant difference in clinical efficacy has been observed between these treatment methods [6, 27, 28]. Radiotherapy is mainly a palliative treatment that relieves symptoms caused by local metastases; however, the patient’s prognosis is very poor, regardless of the selected treatment method. Most affected patients will die within a few months.

As noted, patients with extracranial metastases of malignant glioma have a very poor prognosis and a life expectancy of less than 6 months [32, 33]. The two patients described in this report died within 2 months after the diagnosis of extracranial metastases. From our review of the literature, we determined that the average interval between the diagnosis of extracranial metastases and death was only 4.4 months [6]. The interval between the diagnosis of intracranial lesions and that of extracranial metastasis ranged from 1 to 60 months [2, 17]. Lun et al. [2] reported that for a primary glioblastoma, the interval from the onset of symptoms to diagnosis of the primary glioblastoma was 2.5 months, the median interval from diagnosis to the detection of extracranial metastasis was 8.5 months, and the median interval from metastasis to death ranged from 1.5 to 2.8 months [34]. From 1940 to 2009, the interval from the detection of extracranial metastases to death has progressively increased at a rate of 0.7 months per decade (95% confidence interval, 0.5–1.0 month). The use of MRI has correlated with an increase in overall survival, whereas similar correlations have not been observed for age, sex, or primary glioblastoma site [2].

Although surgery is the preferred treatment strategy for malignant glioma, it can damage the blood-brain barrier, thus allowing tumor cells to form extracranial metastases. Therefore, surgeons should carefully ensure the principle of a non-tumor operation and maintain an intact dura. As described in the literature [33], adherence to the following principles may eliminate or reduce extracranial metastases of malignant gliomas: (1) In addition to resecting the tumor, the operator should aim to protect and reduce damage to the surrounding tissue and should strive for a tumor-free operation, thus strengthening the concept of preventing tumor metastasis. (2) The operator should rinse the surgical field with normal saline and/or chemotherapy drugs and carefully suture the dura to maintain its integrity. (3) Patients with a high degree of malignancy should be treated with radiotherapy and/or chemotherapy. (4) Patients can receive ventriculoperitoneal shunts fitted with filter devices to reduce the implantation intraperitoneal metastases via shunts. (5) Finally, postoperative concurrent chemoradiotherapy may reduce the chance of intracranial recurrence and extracranial metastasis.

## Conclusions

In summary, the glioblastoma might undergo extracranial metastasis. Although we have described extracranial metastases of malignant gliomas in detail, the complex mechanism underlying metastasis remains unclear. Since the metastasis is not sensitive to chemoradiotherapy, the operation should strictly adhere to the tumor-free principle and try to guarantee completeness of cerebral dura mater for the sake of reducing the occurrence rate of extracranial metastasis. Technological advances in the diagnosis and treatment of malignant gliomas and gradual increases in patient survival may lead to a gradual increase in the incidence of extracranial metastases in the future. Currently, however, patients with extracranial metastases of malignant glioma have a poor prognosis, and most die within 6 months of diagnosis.

## Abbreviations

CNS: Central nervous system
CT: Computed tomography
MRI: Magnetic resonance imaging
PET-CT: Positron emission tomography computed tomography
SPECT: Single-photon emission computed tomography
